# Antiviral and Antibacterial 3D-Printed Products Functionalised with Poly(hexamethylene biguanide)

**DOI:** 10.3390/polym16030312

**Published:** 2024-01-23

**Authors:** Anson M. Y. Luk, Chris K. Y. Lo, Jiachi Amber Chiou, Chi-Hang Ngai, Ki Law, Tsz-Long Lau, Wan-Xue Chen, Matthew Hui, Chi-Wai Kan

**Affiliations:** 1 School of Fashion and Textiles, The Hong Kong Polytechnic University, Hung Hom, Kowloon, Hong Kong SAR, China; anson-m-y.luk@polyu.edu.hk (A.M.Y.L.); kwan.yu.lo@polyu.edu.hk (C.K.Y.L.); wanxue.chen@connect.polyu.hk (W.-X.C.); 2 Immune Materials Limited, Room 05, Unit 107-109, 1/F, 9 Science Park West Avenue, Hong Kong Science Park, Pak Shek Kok, N.T., Hong Kong SAR, China; lokilaw@immunematerials.com (K.L.); lau.tszlong0307@gmail.com (T.-L.L.);; 3 Department of Food Science and Nutrition, The Hong Kong Polytechnic University, Hung Hom, Kowloon, Hong Kong SAR, China; jiachi.amber.chiou@polyu.edu.hk; 4 University Research Facility in 3D Printing, The Hong Kong Polytechnic University, Hung Hom, Kowloon, Hong Kong SAR, China; stanley.ngai@polyu.edu.hk

**Keywords:** 3D-printed plastics, additive manufacturing, antibacterial, antiviral, digital light processing, poly(hexamethylene biguanide)

## Abstract

Infection prevention and public health are a vital concern worldwide, especially during pandemics such as COVID-19 and seasonal influenza. Frequent manual disinfection and use of chemical spray coatings at public facilities are the typical measures taken to protect people from coronaviruses and other pathogens. However, limitations of human resources and coating durability, as well as the safety of disinfectants used are the major concerns in society during a pandemic. Non-leachable antimicrobial agent poly(hexamethylene biguanide) (PHMB) was mixed into photocurable liquid resins to produce novel and tailor-made covers for public facilities via digital light processing, which is a popular 3D printing technique for satisfactory printing resolution. Potent efficacies of the 3D-printed plastics were achieved in standard antibacterial assessments against *S. aureus*, *E. coli* and *K. pneumoniae*. A total of 99.9% of Human coronavirus 229E was killed after being in contact with the 3D-printed samples (containing the promising PHMB formulation) for two hours. In an eight-week field test in Hong Kong Wetland Park, antibacterial performances of the specially designed 3D-printed covers analysed by environmental swabbing were also found to be satisfactory. With these remarkable outcomes, antimicrobial products prepared by digital light processing 3D printing can be regarded as a reliable solution to long-term infection prevention and control.

## 1. Introduction

Infection prevention and control is a long-term and important issue in our daily life. Everything that we touch in public areas can be a potential media of contact contamination and cross-infection [1,2], especially during times like the COVID-19 pandemic [3] and seasonal influenza spread [4]. To maintain the hygiene of the environment, regular cleaning and disinfection work is carried out in every place around us. However, it is not guaranteed that all the contact surfaces and facilities are always covered by this manual practice in a vast public area [5].

In order to provide a continuous and extensive disinfection of public facilities, antibacterial and antiviral coatings composed of antimicrobial agents are widely applied owing to the convenience and effectiveness. With the advancement in nanoscience, antimicrobial spray coatings for biomedical applications based on polymers or nanocomposites are being developed that have compelling antimicrobial performances [6,7,8,9,10,11,12]. Nevertheless, the long-term efficacy, i.e., durability of antimicrobial coatings applied to different surfaces via chemical spraying, is the main concern of the end users [13,14]. In general, many coatings available on the market have been proven to last between 3 and 6 months under testing conditions in laboratories. It is widely accepted that the lifecycle of antimicrobial coatings is deteriorated by frequent contact (abrasion and scratching), outdoor weathering and usual manual disinfections, and therefore are unable to provide reliable protection [15].

The antimicrobial agents employed in chemical coatings are also a controversial issue. Many studies have shown that metal ions and metal-based substances, including nanoparticles of silver [16], copper oxide [17], zinc oxide [18], titanium dioxide [19] and gold [20] can be used for making potent antimicrobial coatings. However, the cytotoxicity concerns of such antimicrobial metal nanoparticles are likely to be considered a long-term risk for human health and the environment because of possible accumulation in organs and uncontrolled release of metal ions [21,22]. During the COVID-19 pandemic, various coating products were developed using silver ions or silver nanoparticles as the core antimicrobial agents [23]. There are mainly three biocidal mechanisms about silvers that can address the microbes, including (1) bacterial cell wall and viral envelope are punctured by silver ions (Ag^+^) to form pores, in which Ag^+^ actually react with the peptidoglycan (murein) component, and phospholipids and proteins, respectively once Ag^+^ has entered into the microbial structures; (2) cellular respiration is inhibited and metabolic pathways are disrupted to provoke the generation of reactive oxygen species by which the cell can be further destroyed; and (3) DNA and its replication cycle can also be interfered by Ag^+^ [24]. Although these biocidal effects are promising, their similar impacts on human cells should not be ignored [25]. Some studies [26,27,28,29] have proven that the risk of deep penetration of silver nanoparticles or Ag^+^ into human body via skin contact is quite high. Blue-grey discoloration of the skin and eyes by heavy deposition of precipitates of silver may occur. When silver nanoparticles or ions enter the blood and colon of patients, different blood diseases and colon cancer are the consequences.

**Figure 1:**
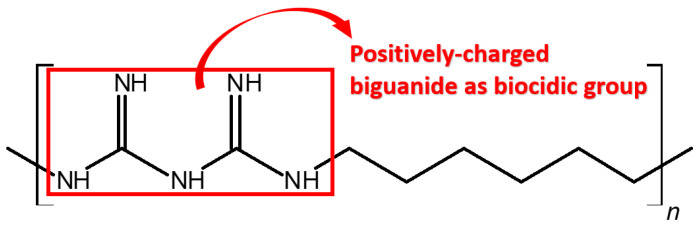
Chemical structure of poly(hexamethylene biguanide).

**Figure 2:**
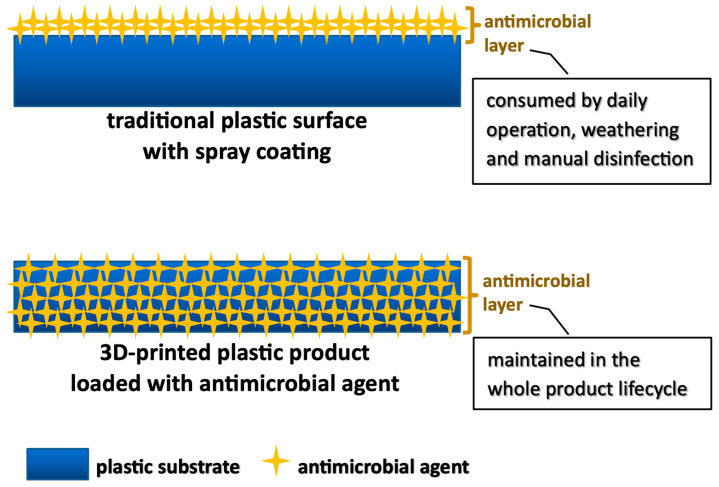
Comparison of antimicrobial plastic products prepared by chemical coating and DLP 3D printing by the cross-sectional views of the plastic products.

Antibacterial and antiviral 3D-printed products were developed to tackle the above challenges for different public facilities. In this study, poly(hexamethylene biguanide) (PHMB), a polymer that bears the positively charged biguanide in the repeating unit of the polymer chain (see Figure 1), was mixed in photosensitive resins to print the antimicrobial samples and products by digital light processing (DLP) technique. PHMB is a mature synthetic antimicrobial agent applied in various industries for over 60 years [30,31,32,33]. Its non-leaching property is attributed to its high molecular weight, approximately 1600 to 2600 g/mol; hence there are many advantages of its applications, such as improved environmental stability, low toxicity, high biocompatibility and satisfactory retention of antimicrobial activity [34,35,36,37,38]. Through DLP photo-curing 3D printing technique [39],

PHMB was evenly dispersed into photosensitive liquid resins, which were cured and solidified under UV during the printing process. This facilitates embedding of the antimicrobial agent in the 3D-printed products layer by layer, instead of being deposited on the surface by spray coating (Figure 2). The entire lifecycle of a material can be covered with the promising antimicrobial efficacies, to provide more complete protection against pathogens. Recently, titanium(IV)-oxo complexes (TOCs) dispersed in various polymer matrices have been studied to develop composite materials with antimicrobial and photocatalytic functions [40,41,42], but such polymeric materials are not applicable to common 3D printing technologies. It is believed that DLP is a highly recommended 3D printing technology for preparing customised plastic products with antimicrobial properties, because of more compatible printing temperature and raw materials to PHMB. The antibacterial efficacies of our 3D-printed samples were evaluated against common pathogens, including *Escherichia coli* (*E. coli*), *Staphylococcus aureus* (*S. aureus*) and *Klebsiella pneumoniae* (*K. pneumoniae*), in laboratory. Antiviral performances against Human coronavirus 229E (HCoV-229E) and biological reactivity test, in vivo, of the 3D-printed materials were assessed by third-party testing laboratories. To have more practical studies, our 3D-printed trial products were examined in field tests and their antibacterial effects were further analysed by surface swab sampling after 8 weeks and assessed by standard tests in laboratory after 19 weeks.

## 2. Materials and Methods

### 2.1. Materials

PHMB ANP-100 (98%), a commercial product of poly(hexamethylene biguanide) hydrochloride with chemical structure (C_8_H_17_N_5_)_n_•xHCl (*n* = 12–16) and molecular weight ≥ 1600~2600 g/mol, was purchased from Guangdong Aona Chemical Industry New Materials Ltd. (Guangzhou, China). Photo-curable 3D-printing resins, E-RigidForm Amber (ERF-A), E-RigidForm Charcoal (ERF-C) and ABS Hi-Impact Gray (ABS-HG) were sourced from EnvisionTEC (Gladbeck, Germany). Bromophenol Blue sodium salt (BPB) is an ACS reagent supplied by Acros Organics (Geel, Belgium). Dimethyl sulfoxide (DMSO) in HPLC grade and distilled water were supplied by Duksan Pure Chemicals Co. Ltd. (Ansan-si, Republic of Korea) and Watson’s Water (Hong Kong, China), respectively for dispersing PHMB powder in the 3D-printing resins. 2-propanol (≥99.5%) for washing 3D-printed products was supplied by Fisher Scientific. Household chlorine bleach was from Kao Corporation (Tokyo, Japan). *Escherichia coli* (*E. coli*, ATCC 8739), *Staphylococcus aureus* (*S. aureus*, ATCC 6538) and *Klebsiella pneumoniae* (*K. pneumoniae*, ATCC 4352) were obtained from the American Type Culture Collection (Manassas, VA, USA).

### 2.2. Fabrications of Antimicrobial Samples and Covers

#### 2.2.1. Preparation of PHMB Solution

First, PHMB stock solution was prepared for mixing with photosensitive 3D-printing resins. A total of 35 g of distilled water was added to 30 g of PHMB powder at 60 °C to prepare the gel mixture. When about half of the PHMB powder was dissolved, 35 g of DMSO was added to the mixture and then stirred gently by a glass rod for 15 min. The resulting mixture was further heated at 60 °C for 5 h to form a well dissolved PHMB solution.

#### 2.2.2. Preparation of Antimicrobial Printing Resin

The 3D-printing resin was pre-mixed by the LC-3DMixer manufactured by 3D Systems (Wilsonville, OR, USA) for 30 min to ensure a uniform resin emulsion. An amount of 30 g of PHMB solution was gently added to 270 g of resin and the PHMB/resin printing mixture was stirred until the PHMB solution and resin were completely miscible. The PHMB/resin printing mixture was then degassed in vacuum oven at −0.08 to −0.1 MPa for 10 min before printing.

#### 2.2.3. Three-Dimensional Printing and Post-Treatments of Antimicrobial Materials

DLP printer, EnvisionTEC Perfactory 4 (Gladbeck, Germany) with a build platform of 160 mm (length) × 100 mm (width) × 230 mm (height), was employed in this study. The antimicrobial covers and samples were printed with a 100-microns-thick layer. Exposure time, UV power, build platform offset and separation speed were set up according to the recommendations of EnvisionTEC. The printed products were immersed in 2-propanol bath for 3 to 5 min to remove the uncured resin and residual chemicals on the surface. Support structures, if present, were also removed by a pair of pliers manually during the immersion. The washed products were dried with clean tissue paper and compressed air until there was no alcohol trapped in the products. Such clean 3D-printed products were finally placed in LC-3DPrint Box installed by 3D Systems (Wilsonville, OR, USA) for 120 min to ensure full curing. The surface of cured products was cleaned with fresh 2-propanol once.

#### 2.2.4. Preparation of Abraded and/or Bleach-Rubbed Samples

For preparing the abraded samples, the post-cured and cleaned plastics were fixed on the flat surface with tape and surface on one side of each was abraded by 1200-grit or 1600-grit sandpaper installed on a sandpaper frame in the forward and backward direction for 3 min. For the samples cleaned with household bleach, the plastic pieces were fixed on the flat surface with tape and surface on one side of each was rubbed with cotton cloth soaked with household chlorine bleach solution (1:49 *v*:*v*, Kao bleach:water) in the forward and backward direction for 5 s three times. For samples treated with both conditions, the plastic pieces were cleaned with household bleach followed by being abraded by 1200-grit or 1600-grit sandpaper according to the same treatment details.

### 2.3. Characterisation of 3D-Printed Samples Doped with PHMB by Decolourisation of BPB Solution

BPB aqueous solution was decolourised by ERF-A 3D-printed sample plates (50 mm × 50 mm × 4 mm) of pristine and with 1, 2 and 3% PHMB for proving the cationic property of the 3D-printed products. One piece of sample plate was immersed into 160 mL of BPB solution (31.3 mg/L in deionized water) in a 250 mL borosilicate glass beaker (sealed with a piece of plastic film) under quick and continuous shaking by orbital shaker (speed: 80 rounds per minute) in 120 min at room temperature. At every 60 min, 5 mL of BPB solution was extracted using micropipette and transferred into a test tube containing 8 mL of deionised water for dilution. The absorption spectrum, 250 to 700 nm, of the diluted BPB solution was obtained by a double beam UV-Vis spectrophotometer, UH5300, manufactured by Hitachi (Chiyoda, Japan).

The absorbance intensities at 0 min (*I*_0_) and different immersion duration (*I_t_*) were recorded for plotting the decolourisation curves by the corresponding sample plates. *I*_0_ of each sample was recorded after sample stabilisation in BPB solution for 5 min. Absorbance of BPB solution decolourised by ERF-A samples of pristine and with 1% PHMB was monitored at 592 nm; while that by ERF-A samples of 2 and 3% PHMB was monitored at 603 nm for plotting decolourisation curves (*I_t_*/*I*_0_ against immersion time).

### 2.4. Quantitative Assessments of Antimicrobial Efficacies of 3D-Printed Samples in Lab Tests

#### 2.4.1. Antibacterial Activities by ISO 22196:2011

Antibacterial test samples (50 mm × 50 mm × 4 mm) printed with ERF-A resin mixed with 0, 2 and 3% PHMB were evaluated for efficacy against Gram-negative *E. coli* and Gram-positive *S. aureus* according to ISO 22196:2011 [43]. The printed, post-cured and alcohol-washed test samples were post-treated with sandpaper abrasion and surface cleaning with a household bleach solution to further examine stability against the simulated daily uses following the same treatment details as mentioned in Section 2.2.4.

Stock solution of *S. aureus* stored at −80 °C was inoculated into 3 mL of sterile tryptone soya broth (TSB) in a 15 mL tube and cultured at 37 °C in bacteria culture room for 24 h. *E. coli* were cultured in same way but using nutrient broth (NB). After overnight culturing, sterile TSB and NB were added separately into *S. aureus* and *E. coli* in order to dilute the bacterial solution to an optical density of 1, measured by a spectrophotometer at 600 nm. Therefore, the final concentration of the bacteria solution was around 3 × 10^8^ CFU/mL. The bacterial solution was then diluted to 1 × 10^6^ CFU/mL as the test inoculum.

Samples were put in a sterile Petri dish, with the tested surface uppermost. A total of 400 µL of the inoculum was added onto the test surface and covered with a piece of film (40 mm × 40 mm). It was ensured that the inoculum does not leak beyond the edges of the film and is spread evenly on the test surface. After inoculation immediately, bacteria in half of the treated and untreated samples were recovered by adding 10 mL of Soya Casein Digest Lecithin Polysorbate (SCDLP) broth. The recovery rate of the bacteria could be calculated by this step. The other samples were cultured at 37 °C in a box with a relative humidity not less than 90% for 24 h.

To determine the viable bacteria recovered from the tested samples, 10-fold serial dilutions of the SCDLP in phosphate-buffered physiological saline (PBS) were collected and 1 mL of each dilution was mixed with 15 mL of plate count soft agar in Petri dish in triplicate. Plate was cultured at 37 °C for 24 h and clear colonies were counted and recorded. The CFU/mL values, which indicate levels of microorganisms, i.e., *E. coli* and *S. aureus* on the sample surfaces in this study, were calculated using the method of aerobic plate count following Equation (1), according to the Bacteriological Analytical Manual 2001 published by U.S. Food and Drug Administration:

*N* = (∑*C*)/{[(1 × *n*_1_) + (0.1 × *n*_2_)] × *d*} (1)

where *N* = number of colonies per mL or g of product; Σ*C* = sum of all colonies on all plates counted; *n*_1_ = number of plates in first dilution counted; *n*_2_ = number of plates in second dilution counted; and *d* = dilution from which the first counts were obtained.

#### 2.4.2. Inhibition Zone Determination by AATCC TM 147:2011

Inhibition zones against *E. coli*, *S. aureus* and *K. pneumoniae* streak cultures were identified by our 3D printing formulations, in accordance with AATCC TM 147:2011 [44]. Plastic test samples (50 mm × 50 mm × 4 mm) were prepared with ERF-A resin loaded with 0, 1, 2, 3 and 4% PHMB; ERF-C resin added with 0, 1 and 3% PHMB. The selected printed test samples were post-treated with sandpaper abrasion and surface cleaning by a household bleach solution to further examine the stability against the simulated daily uses according to the same treatment details as mentioned in Section 2.2.4.

Overnight-cultured *S. aureus* and *E. coli* were diluted with sterile TSB and NB until the optical density was equal to 1 at 600 nm. Five lines of cultured bacteria were streaked on the surface of tryptone soya agar (TSA) or nutrient agar (NA) separately; the samples were then placed to attach to streaks. The test samples were transversely pressed across the five inoculum streaks to ensure intimate contact with the agar surface. After being cultured at 37 °C for 24 h, widths of clear zone of inhibition were measured. The interruption of growth along the streaks of inoculum beneath the sample can be examined by this evaluation.

#### 2.4.3. Antiviral Performances by ISO 21702:2019

Antiviral test samples (50 mm × 50 mm × 3.4 mm) printed with ERF-A resin mixed with 0 and 3% PHMB were examined for efficacy against Human coronavirus 229E (HCoV-229E) by a third-party testing lab, Guangdong Detection Centre of Microbiology, according to ISO 21702:2019 [45].

### 2.5. Biological Reactivity Test, In Vivo, by USP General Chapter <88>

A test sample (50 mm × 50 mm × 3.4 mm, 10.6 g) printed with ERF-A resin mixed with 3% PHMB was evaluated for intradermal reactivity in rabbit by a third-party testing lab, Hong Kong Standards and Testing Centre (Dongguan), according to the standard method provided by United States Pharmacopeia (USP), General Chapter <88>, Class I.

### 2.6. Quantitative Examination of Antibacterial Effectiveness of 3D-Printed Covers by Field Test

**Figure 3:**
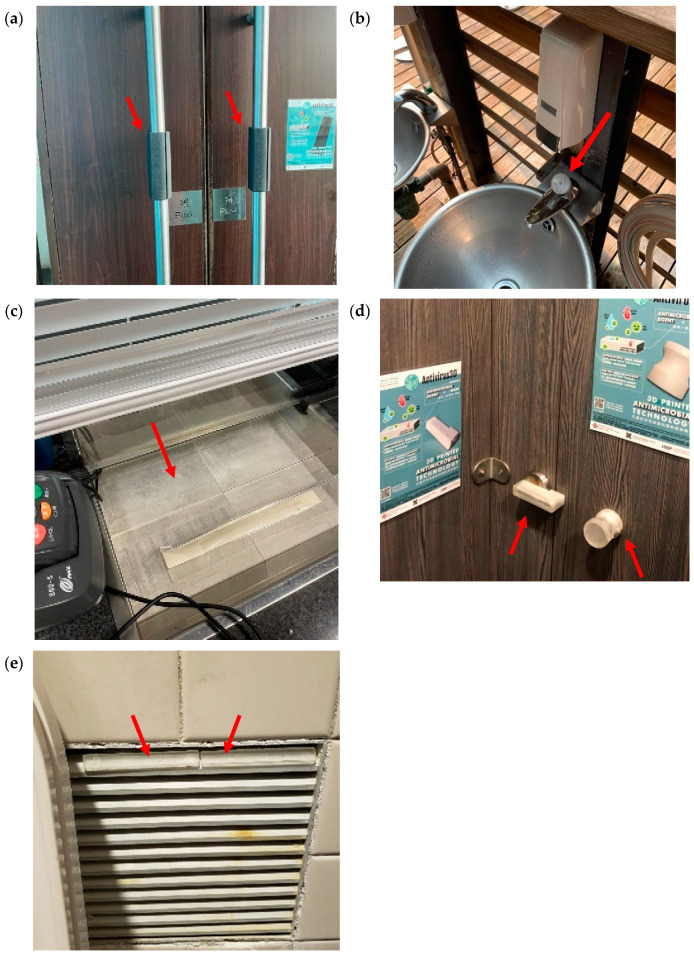
Photos of facilities covered with 3D printing products prepared with PHMB formulations installed in Hong Kong Wetland Park (indicated by red arrows); (**a**) door’s pulling handles of exhibition hall on the 1/F; (**b**) push button of faucet at the park entrance; (**c**) surface of stainless-steel tray at ticket counter; (**d**) door lock of toilet cubicle in males’ toilet on the G/F; and (**e**) ventilation window in toilet cubicle in men’s toilet on the G/F.

From 9 March to 4 May 2021, during the fourth wave of the COVID-19 pandemic (late November 2020 to late May 2021) in Hong Kong [46], a field test trial was conducted for eight weeks to examine the antibacterial performance of our 3D-printed covers fabricated with 3% PHMB formulation in Hong Kong Wetland Park. As presented in Figure 3, door handles, faucet button, surface of the stainless-steel tray at ticket counter and door lock of the toilet cubicle were covered by the antimicrobial 3D-printed covers. Antimicrobial 3D-printed bar samples (80 mm × 10 mm × 5 mm) were also stuck on the ventilation window in toilet cubicle for evaluating their antibacterial performances. All of these covers were made of ERF-A or ERF-C.

After eight weeks of exposure in real practice, the covered and uncovered surfaces (as controls) were sampled using cotton swab kits supplied by SGS Hong Kong Ltd (Hong Kong SAR, China). Sampled swabs, stored at 0 to 4 °C were delivered to the testing lab of SGS Hong Kong Ltd. within 24 h for analysis. The total bacteria count of covered and control surfaces was determined by AOAC official method 18th ed., 2005, Method 990.12; counts of *E. coli* and *S. aureus* of the covered surfaces were measured as per AOAC official method 18th ed., 2005, Method No. 991.14 and 2006, Method No. 2003.07/08/11, respectively.

After the field test trial for 19 weeks, the 3D-printed covers installed in Hong Kong Wetland Park were brought back to the laboratory on 20 July 2021. Covers for stainless-steel tray at ticket counter, door lock of toilet cubicle and bars on ventilation window in toilet cubicle were selected as specimens for further antibacterial assessments. The surfaces of these used covers were sprayed with a 70% ethanol solution and dried with clean tissue paper. The cleaned covers were fragmented into small and flat pieces of 50 mm × 50 mm (width × length), to evaluate the remaining efficacy against Gram-negative *E. coli* and Gram-positive *S. aureus* according to ISO 22196:2011 [43], following the experimental details in Section 2.4.1. The tested surfaces of certain selected cover pieces for stainless-steel tray at ticket counter, door lock of toilet cubicle and bars on ventilation window in toilet cubicle were also abraded by 1000-grit sandpaper in the forward and backward direction for 10 s (10–12 turns) manually, in order to further evaluate their antibacterial efficacy after being abraded. The CFU/mL values, which indicate the levels of microorganisms, i.e., *E. coli* and *S. aureus* on the sample surfaces in this study, were calculated using the aerobic plate count following Equation (1).

## 3. Results and Discussion

### 3.1. Characterisation of Antimicrobial 3D-Printed Samples by Decolourisation of BPB Solution

**Figure 4:**
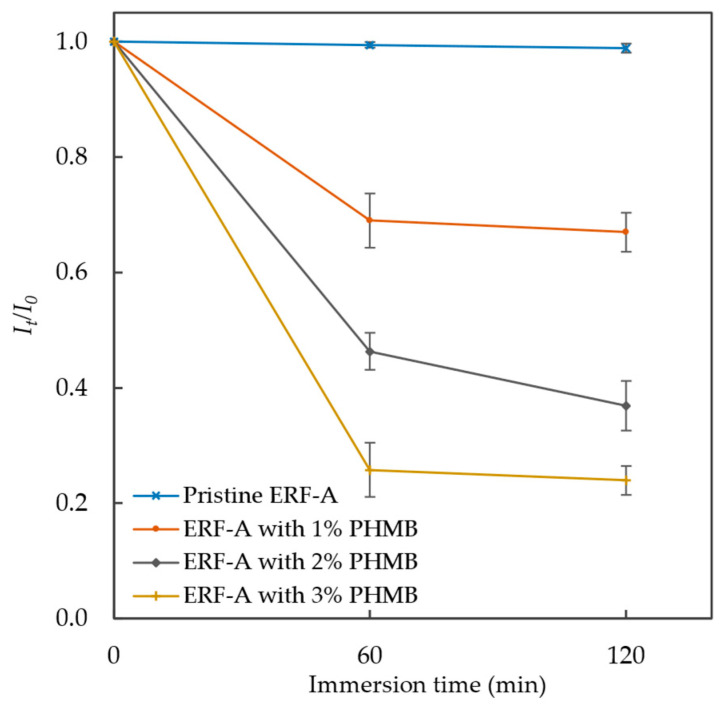
Decolourisation of BPB solution (31.3 mg/L) by 3D-printed ERF-A sample plates doped with different % contents of PHMB (presented with error bars of standard deviations).

In previous studies [33,47], BPB, which is an anionic dye complexed with cationic agents at room temperature by electrostatic attraction, can be used for quantifying the PHMB coated onto cotton fabric. The decolourisation extents of BPB aqueous solution are demonstrated in Figure 4 to characterise the cationic nature of the 3D-printed ERF-A materials doped with different % contents of PHMB. The rates of BPB decolourisation were proportional to the PHMB contents in ERF-A sample plates to indicate the extent of cationic properties of the 3D-printed materials contributed by PHMB.

### 3.2. Antibacterial Performances against E. coli and S. aureus by ISO 22196

The efficacies of the 3D-printed samples, ERF-A, with 0, 2 and 3% (*w*/*w*) PHMB against *E. coli* and *S. aureus* were evaluated (Table 1). The samples without PHMB are the control samples that demonstrate that there were no antibacterial effects of the pure ERF-A material. With addition of 2% PHMB to the resin, the printed samples were able to perform instant contact-killing of *S. aureus* and *E. coli* at 0 h (The viable bacteria count of the bacteria to be recovered from the test specimen immediately after inoculation was determined after incubation at (35 ± 1) °C for 40 h to 48 h.); meanwhile, complete reduction of both bacteria could be finally achieved after 24 h. The same antibacterial effects were also demonstrated by the ERF-A plastic pieces with higher PHMB content, i.e., 3% *w*/*w*.

To further assess the stability of the 3D-printed samples in daily use, three surface post-treatments of ERF-A samples, which were (1) abraded with sandpaper (2) cleaned with 1:49 household chlorine bleach solution; and (3) cleaned with 1:49 household chlorine bleach solution followed by abrasion with sandpaper were performed. The regular cleaning and usage routines in public area, in which antibacterial surface coating cannot last, were mimicked. As presented in Table 1, excellent instant contact-killing and inhibition of *E. coli* after 24 h were demonstrated by the abraded ERF-A samples with 2% and 3% PHMB contents. While instant contact-killing of *S. aureus* could not be realised, potent disinfecting power was still exhibited by the PHMB after 24 h. For ERF-A samples pre-rubbed with 1:49 household chlorine bleach solution (strong oxidising agent), promising instant contact-killing and inhibition effects, especially for the ones containing 3% PHMB, could still be preserved. PHMB agent was stably accommodated in ERF-A matrix against an oxidative attack by chlorine bleach. When the ERF-A samples were pre-rubbed with 1:49 chlorine bleach solution followed by abrasion with sandpaper, it is surprising that immediate contact-killing effect to *E. coli* could be exhibited by the samples with 2% PHMB only, but not with 3% PHMB. A possible explanation is that ERF-A samples containing higher PHMB content are mechanically softer. The higher content of antimicrobial agent implies a higher chance of being lost by both the chemical attack and physical abrasion; therefore, the contact-killing power of the 3% PHMB/ERF-A samples would be weakened to a certain extent. Nevertheless, remarkable disinfecting efficacies of the ERF-A samples with PHMB were still maintained after 24 h incubation.

### 3.3. Comparison of Inhibition Zones Formed against E. coli and S. aureus by AATCC 147

**Figure 5:**
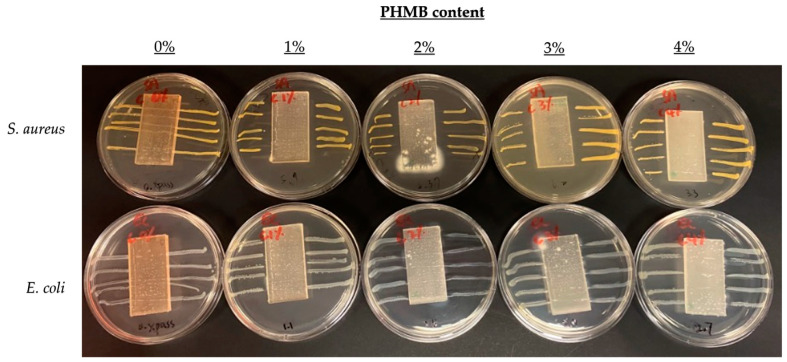
Images of *S. aureus* and *E. coli* streak cultures after 24 h incubation with ERF-A samples loaded with 0% to 4% PHMB without post-treatment.

**Figure 6:**
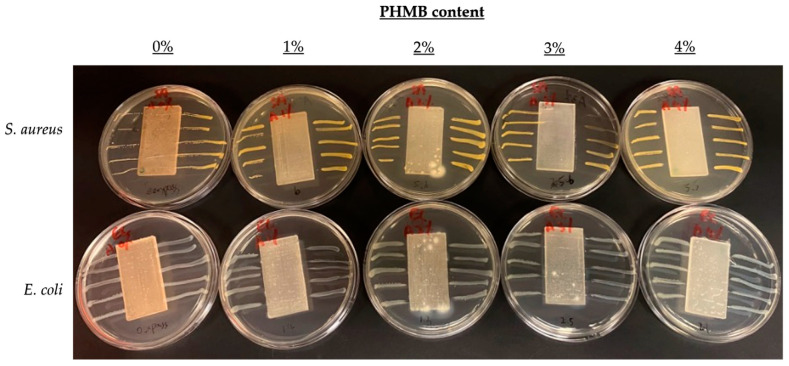
Images of *S. aureus* and *E. coli* streak cultures after 24 h incubation with ERF-A samples loaded with 0% to 4% PHMB abraded with sandpaper.

**Figure 7:**
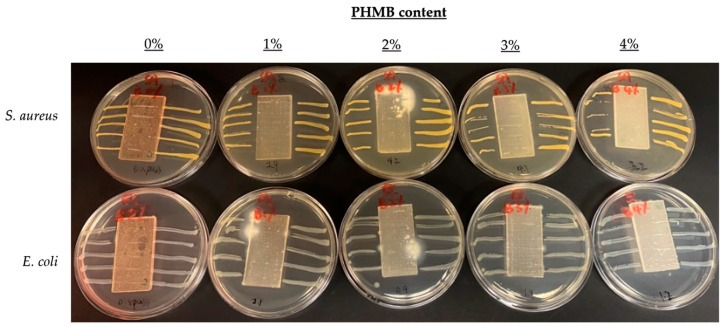
Images of *S. aureus* and *E. coli* streak cultures after 24 h incubation with ERF-A samples loaded with 0% to 4% PHMB cleaned with 1:49 household bleach solution.

**Figure 8:**
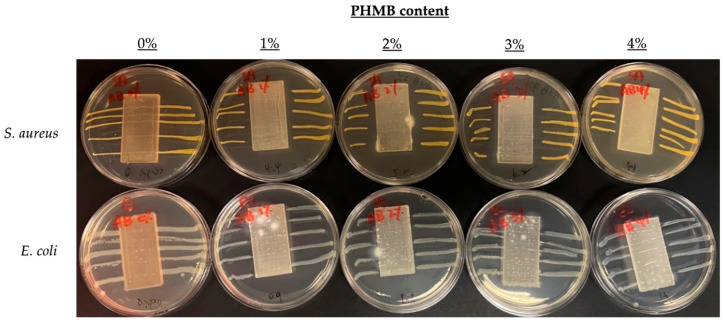
Images of *S. aureus* and *E. coli* streak cultures after 24 h incubation with ERF-A samples loaded with 0% to 4% PHMB cleaned with 1:49 household bleach solution, followed by being abraded with sandpaper.

**Figure 9:**
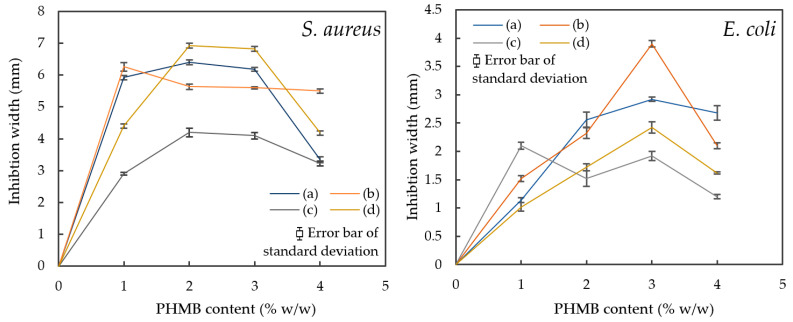
Formation of inhibition widths of various ERF-A samples loaded with 0% to 4% against *S. aureus* and *E. coli* streak cultures after incubation: samples (a) without post-treatment; (b) abraded with sandpaper; (c) cleaned with 1:49 household bleach solution and (d) cleaned with 1:49 household bleach solution, followed by being abraded with sandpaper.

To further compare the effect of PHMB content on antibacterial efficacy, another quantitative antibacterial assessment was conducted to determine the inhibition zones formed by the ERF-A samples loaded with varying contents of PHMB, according to the AATCC TM 147 [44]. The inhibition zones of the ERF-A/PHMB samples with different post-treatments are shown in Figure 5, Figure 6, Figure 7 and Figure 8. The widths of inhibition zones established by different ERF-A/PHMB samples against *S. aureus* and *E. coli* streak cultures after incubation are summarised in Figure 9. Upon the increase of PHMB loading in the ERF-A plastics from 0% to 3% *w*/*w*, wider inhibition zones were generally formed against *S. aureus* and *E. coli* streaks owing to more antimicrobial agents being present. When the ERF-A plastics were prepared with 4% PHMB content, both the inhibition widths towards both S. *aureus* and *E. coli* streaks were reduced. Such diminished efficacies in bacterial inhibition possibly resulted from considerable agglomeration of PHMB polymers in the 4% PHMB samples. ERF-A plastics cleaned with 1:49 household bleach solution probably were relatively weaker against *E. coli* than the others. Although the performances of the ones loaded with 2% and 3% PHMB were already lowered, acceptable inhibition width (not narrower than 1.5 mm) could still be achieved.

### 3.4. Inhibition Zones Formed against K. pneumoniae and S. aureus by AATCC 147

**Figure 10:**
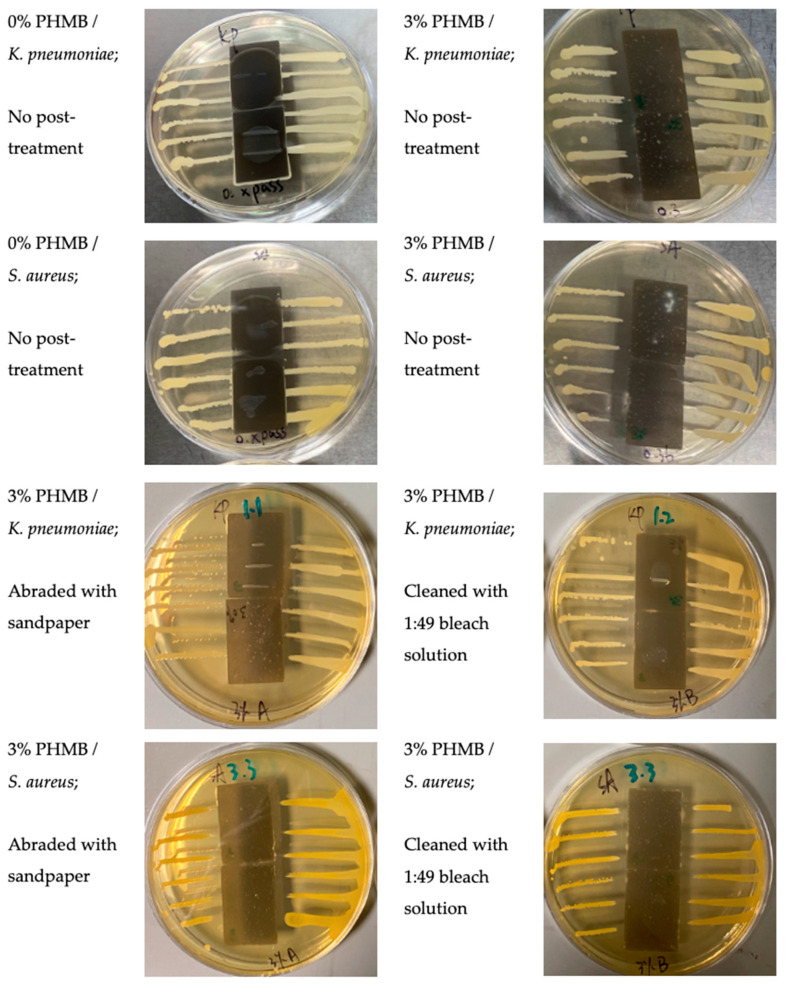
Images of *K. pneumoniae* and *S. aureus* streak cultures after incubation with ERF-C samples loaded with 0% and 3% PHMB contents under different post-treatment conditions.

As the door handles of exhibition hall on 1/F in Hong Kong Wetland Park (see Figure 3a) were made of ERF-C material, the 3D-printed test samples were also prepared with ERF-C/PHMB matrices. Both ERF-A and ERF-C were made of the same resin material but in different colours. Therefore, antibacterial performances of ERF-C samples were assessed against *S. aureus* and another Gram-negative bacterium, *K*. *pneumoniae*. The inhibition effects to *K*. *pneumoniae* and *S. aureus* streak cultures by ERF-C samples are summarised in Table 2 and Figure 10. Effective antibacterial performances demonstrated inhibition of *K*. *pneumoniae* and *S. aureus* by the ERF-C samples mixed with 3% PHMB. The surface of ERF-C (with 3% PHMB) against *K*. *pneumoniae* tended to be lightened by abrasion with sandpaper and cleaning with 1:49 chlorine bleach solution.

### 3.5. Antiviral Activity of 3D-Printed Samples by ISO 21702

HCoV-229E is another strain of human Coronavirus (strain 229E) that shares a strong family design with the outbreak strain, with only minor differences in RNA when compared to the new strain of human Coronavirus, SARS-CoV-2 [48]. If the testing against HCoV-229E can be performed and passed by the test specimen, this may suggest the formulation of the test specimen is effective against the SARS-CoV-2 as well [49]. A sample achieving an antiviral activity value (R-value) of 1 can be considered to bear minimal antiviral property, i.e., an antiviral activity rate of 90.0%, while satisfactory antiviral performance is generally represented by the R-value of 2 in the market. As indicated in Table 3, R-value of ERF-A with 3% PHMB was 3, which means a three-fold increase compared to the reference sample. A total of 99.9% antiviral activity rate against the HCoV-229E virus could be achieved in 2 h. ERF-A plastic without PHMB had the R-value of 1.05, which means limited antiviral activity. It may be because viral survival was not favoured by the smooth surface of our high-density 3D-printed substrate.

### 3.6. Biological Reactivity Test, In Vivo, by USP General Chapter <88>

If plastics are classified based on end use, type and time of exposure of human tissue to plastics by USP, the 3D-printed products designed for covering the frequently contacted areas in public facilities are classified as USP Class I (surface devices for contact to skin). As indicated in Table 4, there was no significant irritation from the sodium chloride extracts injected intracutaneously into rabbits. The mean and mean difference of the erythema and edema scores were also reported to be 0.0 in the test report DY22040369 issued by Hong Kong Standards and Testing Centre (Dongguan, China). The ingredients in our antimicrobial 3D-printed material were proven not to cause skin irradiation to the end users.

### 3.7. Antibacterial Activities of 3D-Printed Covers in Hong Kong Wetland Park

Table 5 shows that the total bacteria count of the facilities covered with our tailor-made 3D-printed covers, which were printed with 3% effective content of PHMB, was significantly lower than those uncovered (controls) in Hong Kong Wetland Park. In addition, these surfaces were also sampled with swabs to determine that there were less than 5 CFU/mL of *S. aureus* and *E. coli* on the covered facilities. They were significantly below the threshold for being pathogenic.

As shown in Table 6, the 3D-printed sample collected after 19 weeks of installation retains its antibacterial properties. Instant contact-killing and 24 h inhibition towards *E. coli* and *S. aureus* were demonstrated potently by toilet door locks, which are a high-risk infection vector. Contact-killing towards both *E. coli* and *S. aureus* could not be provided by some of the used covers. Nevertheless, complete and outstanding inhibition could still be achieved within 24 h. It is believed that our designs and developments were effective in preventing the covered facilities in Hong Kong Wetland Park from being the infection media for microbes.

## 4. Conclusions

Novel antibacterial and antiviral plastic materials are successfully developed with DLP 3D-printing technique. Extensive assessments of antibacterial properties against *S*. *aureus*, *E. coli* and *K*. *pneumoniae*, as well as an antiviral test against HCoV-229E show that the 3D-printing photosensitive resins mixed with 3% PHMB are promising and provide effective and safe prevention of pathogenic infections. During field testing in Hong Kong Wetland Park, significant inhibition of bacterial growth was observed, which is considered compelling evidence of the antimicrobial efficacies of our designs and developments in real-world practice. Further investigations should study the antimicrobial performances in different field environments.

The substantial potential of DLP techniques in developing 3D-printed products with convincing antibacterial and antiviral performances is highlighted by the outcomes reported in this study. Notable contributions have been made in the fields of antimicrobial technology and infection prevention and control.

## Figures and Tables

**Table 1:** CFU/mL values of *S. aureus* and *E. coli* on the surfaces of ERF-A samples, which were printed with different PHMB contents and treated with different surface post-treatments, at 0 and 24 h (CFU/mL values concluded by aerobic plate counts according to Bacteriological Analytical Manual 2001 published by U.S. Food and Drug Administration (Maryland, United States)).

		Bacteria Contact Hour			
Post-Treatment		*S. aureus* (CFU/mL)		*E. coli* (CFU/mL)	
	PHMB % *w*/*w*	0	24	0	24
No treatment	0	1.72 × 10^6^	2.21 × 10^9^	4.10 × 10^5^	2.59 × 10^10^
	2	0	0	0	0
	3	0	0	0	0
Abraded with sandpaper	0	1.94 × 10^6^	1.85 × 10^9^	6.80 × 10^6^	8.82 × 10^10^
	2	1.13 × 10^6^	0	0	0
	3	2.05 × 10^6^	0	0	0
Cleaned with 1:49 household bleach solution	0	1.96 × 10^6^	2.17 × 10^8^	1.23 × 10^6^	9.18 × 10^9^
	2	1.40 × 10^6^	0	0	0
	3	0	0	0	0
Cleaned with 1:49 household bleach solution and then abraded with sandpaper	0	1.66 × 10^6^	2.69 × 10^8^	1.05 × 10^9^	1.34 × 10^12^
	2	0	0	0	0
	3	0	0	6.80 × 10^5^	0

**Table 2:** Inhibition widths, in mm, of ERF-C plastics with different PHMB contents toward *K. pneumoniae* and *S. aureus* streak cultures under different post-treatment conditions.

	*K. pneumoniae*			*S. aureus*		
PHMB Content (% *w*/*w*)	Without Post-Treatment	Abraded with Sandpaper	Cleaned with 1:49 Bleach Solution	Without Post-Treatment	Abraded with Sandpaper	Cleaned with 1:49 Bleach Solution
0	0	0	0	0	0	0
3	3.0	1.1	1.2	3.6	3.3	3.3

**Table 3:** Antiviral activity of ERF-A samples with 0% and 3% PHMB against HCoV-229E (retrieved from test reports 2021FM09338R01E and 2021FM09338R02E by Guangdong Detection Centre of Microbiology).

	Infectivity Titre Value Immediate after Inoculation of the Reference Specimen	Infectivity Titre Value after 2 h Contacting with the Reference Specimen	Infectivity Titre Value after 2 h Contacting with the ERF-A Sample with 0% PHMB	Infectivity Titre Value after 2 h Contacting with the ERF-A Sample with 3% PHMB
Average logarithm of infectivity titre value (lgTCID_50_/mL)	5.81	5.17	4.12	2.17
Average logarithm of infectivity titre value (lgTCID_50_/cm^2^)	5.61	4.97	3.92	1.97
Average infectivity titre value (lgTCID_50_/cm^2^)	4.07 × 10^5^	9.35 × 10^4^	8.33 × 10^3^	93.50
Antiviral activity value (R-value)	-	-	1.05	3.00
Antiviral activity rate (%)	-	-	91.1	99.9

**Table 4:** USP intracutaneous observations of ERF-A samples with 3% PHMB (retrieved from test report DY22040369 by Hong Kong Standards and Testing Centre (Dongguan)).

Animal Number	Sodium Chloride Solution Extract	Scoring Interval (Erythema Site/Edema Site)			
		Immediately after Injection	24 h	48 h	72 h
Rabbit A	From ERF-A with 3% PHMB	0/0	0/0	0/0	0/0
	From control	0/0	0/0	0/0	0/0
Rabbit B	From ERF-A with 3% PHMB	0/0	0/0	0/0	0/0
	From control	0/0	0/0	0/0	0/0

Definition of test scoring: 0: No erythema; No edema; 1: Very slight erythema (barely perceptible); Very slight edema (barely perceptible); 2: Well-defined erythema; Well-defined edema (edges of area well-defined by definite raising); 3: Moderate erythema; Moderate edema (raised approximately 1 mm); 4: Severe erythema (beet redness) to eschar formation preventing grading of erythema; Severe edema (raised more than 1 mm and extending beyond exposure area).

**Table 5:** Swab results of total bacteria counts of the surface of ERF-A or ERF-C covers prepared with 3% *w*/*w* PHMB for facilities in Hong Kong Wetland Park (retrieved from test reports HKIEQ21-00083 R0 and HKIEQ21-00111 R0 by SGS Hong Kong Ltd.).

Facility	Control	3D-Printed Cover	Inhibitory
	(CFU/mL)	(CFU/mL)	(%)
Door’s pulling handle of exhibition hall on the 1/F	45	2	95.6
Push button of faucet at the park entrance	16,000	14	99.9
Surface of stainless-steel tray at ticket counter	520	78	85.0
Door lock of toilet cubicle in males’ toilet on the G/F	183.5	<1	≥99.5
Ventilation window in toilet cubicle in males’ toilet on the G/F	130	7	94.6

**Table 6:** CFU/mL values of *S. aureus* and *E. coli* on different surfaces of ERF-A covers, produced with 3% *w*/*w* PHMB and applied for 19 weeks in Hong Kong Wetland Park, at 0 and 24 h (CFU/mL values concluded by aerobic plate counts according to the method in Bacteriological Analytical Manual 2001 published by U.S. Food and Drug Administration).

	Contact Hour of Bacteria			
Sample	*S. aureus* (CFU/mL)		*E. coli* (CFU/mL)	
	0 h	24 h	0 h	24 h
Covers for ticket counter (Normal)	0	0	0	0
Covers for ticket counter (Abraded)	0	0	4.10 × 10^7^	0
Door lock of toilet cubicle (Normal)	0	0	0	0
Door lock of toilet cubicle (Abraded)	0	0	0	0
Bar on ventilation window (Normal)	1.31 × 10^6^	0	3.10 × 10^9^	0
Bar on ventilation window (Abraded)	1.21 × 10^6^	0	4.50 × 10^4^	0

## Data Availability

The data presented in this study are available on request from the corresponding author (privacy and legal).
